# A Higher Correlation of HCV Core Antigen with CD4+ T Cell Counts Compared with HCV RNA in HCV/HIV-1 Coinfected Patients

**DOI:** 10.1371/journal.pone.0023550

**Published:** 2011-08-12

**Authors:** Tao Shen, Xiangmei Chen, Weidong Zhang, Yuanlin Xi, Guanghua Cao, Yuhong Zhi, Shuiwang Wang, Chunhui Xu, Lai Wei, Fengmin Lu, Hui Zhuang

**Affiliations:** 1 Department of Microbiology and Infectious Disease Center, Peking University Health Science Center, Beijing, China; 2 Department of Epidemiology, College of Public Health, Zhengzhou University, Zhengzhou, Henan, China; 3 Shangcai County People's Hospital, Shangcai, Henan, China; 4 Shangcai Center for Disease Control and Prevention, Shangcai, Henan, China; 5 Institute of Hepatology, Peking University People's Hospital, Beijing, China; University of Toronto, Canada

## Abstract

Development of HCV infection is typically followed by chronic hepatitis C (CHC) in most patients, while spontaneous HCV viral clearance (SVC) occurs in only a minority of subjects. Compared with the widespread application of HCV RNA testing by quantitative RT-PCR technique, HCV core antigen detection may be an alternative indicator in the diagnosis of hepatitis C virus infections and in monitoring the status of infectious individuals. However, the correlation and differences between these two indicators in HCV infection need more investigation, especially in patients coinfected by HIV-1. In this study, a total of 354 anti-HCV and/or anti-HIV serum positive residents from a village of central China were enrolled. Besides HCV-related hepatopathic variables including clinical status, ALT, AST, anti-HCV Abs, as well as the altered CD4+/CD8+ T cell counts, HCV core antigen and HCV viral load were also measured. The concentration of serum HCV core antigen was highly correlated with level of HCV RNA in CHC patients with or without HIV-1 coinfection. Of note, HCV core antigen concentration was negatively correlated with CD4+ T cell count, while no correlation was found between HCV RNA level and CD4+ T cell count. Our findings suggested that quantitative detection of plasma HCV core antigen may be an alternative indicator of HCV RNA qPCR assay when evaluating the association between HCV replication and host immune status in HCV/HIV-1 coinfected patients.

## Introduction

Infection of hepatitis C virus (HCV) and human immunodeficiency virus-1 (HIV-1) was prevalent in several provinces of China owing to unsanitary commercial blood collection practices until the end of the 1990's [1]–[3] while HCV is more frequently transmitted through unsanitary blood or blood products, compared to HIV-1infection [4]–[7]. Unlike HIV-1, it was reported that approximately 14% to 40% of people infected with HCV spontaneously cleared the virus and had no detectable serum HCV RNA [8]–[14]. Anti-HCV seropositive individuals with detectable HCV RNA were considered to have active HCV infection and were classified as chronic hepatitis C infection (CHC), while HCV seropositive individuals with HCV RNA negative (i.e., viremia-negative) were considered to have a prior HCV infection and were classified as spontaneous HCV viral clearance (SVC) [15].

With the development of techniques for direct detection of the HCV virus (RNA or core protein), it is expected that HCV infectious status can be evaluated better if the results of HCV antibodies and virus detection were considered together. Of note, compared with the widespread application of HCV RNA detection by using the RT-qPCR technique, the HCV core antigen assay may be a useful aid in the diagnosis of suspected hepatitis C viral infections and to monitor the status of infectious individuals. However, the application and significance of HCV core antigen assay and its correlation with HCV RNA detection are still not well investigated, especially on the background of HIV-1 coinfection.

In this cross-sectional study, we analyzed and compared the serological and virological characteristics of HCV viremia-positive and viremia-negative patients in a total of 354 HCV and/or HIV-1 seropositive subjects. Clinical correlations and the effect of HIV-associated factors on abnormalities of liver function in HCV/HIV-1 coinfected patients were also evaluated. The results demonstrated that serum HCV core antigen testing has comparable sensitivity and highly stability to HCV RNA qPCR in CHC patients with or without HIV-1 coinfection and quantitative detection of plasma HCV core antigen may be a practical alternative to the HCV RNA qPCR assay in clinical evaluation of HCV infection. However, HCV core antigen level was negatively correlated with CD4+ T cell counts and anti-HCV antibody response (S/CO ratio) was positively correlated with CD4+ T cell counts in HIV seropositive CHC patients with CD4+ T cell counts less than 1000/µl, while no correlation was found between HCV RNA level and CD4+ T cell count. Our findings suggested that HCV core antigen probably may be more sensitive to immune pressure than HCV RNA under the immunodeficiency condition induced by HIV-1 coinfection.

## Materials and Methods

### Establishment of a study cohort

A total of 1252 residents (account for 80% of the local population) from a village of Shangcai county, Henan province in central China were investigated for serum HBsAg, anti-HCV antibodies and anti-HIV antibodies existence by local CDC (Shangcai Center for Disease Control and Prevention) and local hospital (Shangcai County People's Hospital). 354 individuals who were anti-HCV seropositive and/or anti-HIV-1 seropositive, but negative for HBsAg, were enrolled in this cross-sectional study in August 2009. None of the participants received any form of HCV antiviral therapy. More than 90% of the participants were so-called former blood donors (FBDs) who have a history of non-standard paid blood donation and others were their parents, spouse or children. All participants were interviewed by trained and qualified staff using a standardized questionnaire, including detailed general information, blood donation history, and usage of antiviral or antiretroviral drugs. Anti-HCV antibodies, HCV RNA level, HCV core antigen level and signs of hepatopathy (clinical status, CD4/CD8 T cell counts, ALT, AST, GGT, alkaline phosphatase, albumin, bilirubin, platelet count and liver ultrasound examination) were measured in all studied subjects. All HIV-1 infected individuals received regularly (∼75%) or intermittently (∼10%) first-line highly active antiretroviral therapy (HAART) regimes consisting of two nucleoside reverse transcriptase inhibitors (NRTIs) AZT/ddI (∼60%) or d4T/3TC (∼40%), and one non-nucleoside reverse transcriptase inhibitor (NNRTI) NVP, which were supported by the China CARES (Community AIDS Resource and Education Services) program. The study was approved by institutional review authorities of Peking University Health Science Center and informed consent forms were signed by all participants. The characteristics of all subjects at enrollment are presented in Table S1. An additional nineteen healthy controls were recruited (HIV and HCV seronegative and HBsAg negative subjects) from the same village (although not included in this study cohort).

### Sample collection and clinical evaluation

Serum and plasma were collected at enrollment. Liver associated enzymes including ALT (alanine aminotransferase), AST (aspartate aminotransferase), γ-GT (γ glutamyl transpeptidase), ALP (alkaline phosphatase), albumin, globulin, and albumin/globulin ratio were measured by traditional clinical standardized methods.

### CD4+/CD8+T-cell counts

CD4+/CD8+ T-cell absolute counts were carried out by standardized single platform counting technologies employing cytometric methods. All reagents were obtained from BD Biosciences (BD Biosciences, San Jose, CA) and CD4+/CD8+ T cell counts were determined within 12 hours by using a FACS Calibur (BD Biosciences, San Jose, CA).

### HCV genotyping

A 573-bp fragment in the Core gene of HCV genome was amplified by semi-nested PCR (first with P1-upper and P3-lower, then with P1-upper and P2-lower). P1-upper: gcgaaaggcctt ctggta; P2-lower: cgcacggcacgcacccggg; P3-lower: ccgcagag(a/g)tc(c/t)cccacgta. All PCR reactions were carried out with negative controls in a designated PCR clean room. PCR products were subject to sequence analysis by cycle-sequencing and dye terminator methods with DNA Sequence Analyzer ABI 3730xl (Applied Biosystems Inc., Foster City, CA).

### HIV seropositive screening

HIV-1 was screened by an ELISA assay (GBI Biotech Co., Ltd., Beijing, China) for HIV antibody, and positive tests were confirmed by HIV Blot 2.2 WB assay (HIV Blot 2.2 WB; Genelabs Diagnostics, Singapore) by the local Center for Disease Control and Prevention.

### Detection of HCV antibodies and viral load

All serum specimens were tested for the presence of anti-HCV antibodies using the ARCHITECT Anti-HCV System (Abbott Diagnostics, Abbott Park, USA). HCV antibodies were considered reactive if the S/CO ratio (sample RLU/cutoff RLU) was greater than or equal to 1.0. All samples with S/CO ratio between 1.0 and 5.0 were confirmed by RIBA assay (HCV BLOT 3.0, MP diagnostics, USA). Plasma HCV viral load was determined with the Abbott RealTime™ HCV Amplification Kit (Abbott Molecular Inc. Des Plaines, IL, USA) according to the manufacturer's instructions. The detection limit is 30 IU/ml, equivalent to 1.48 log_10_IU/ml.

### Quantitative detection of HCV core antigen

Plasma HCV core antigen was quantitated using a commercial chemiluminescent microparticle immunoassay (CMIA) (6L47 ARCHITECT HCV Ag Reagent Kit, Abbott Diagnostics, Abbott Park, USA) according to the manufacturer's instructions, with a detection limit of 3 fmol/l.

### Statistical analysis

Spearman's rank-correlation, Wilcoxon matched-pairs, Mann-Whitney U-tests and Pearson Chi-Square Tests (Yates' correction for continuity is used in certain situations) were performed using GraphPad Prism 5.0 software when necessary.

## Results

### Characteristics of the study participants from Shangcai prefecture, Henan, China

**Figure 1:**
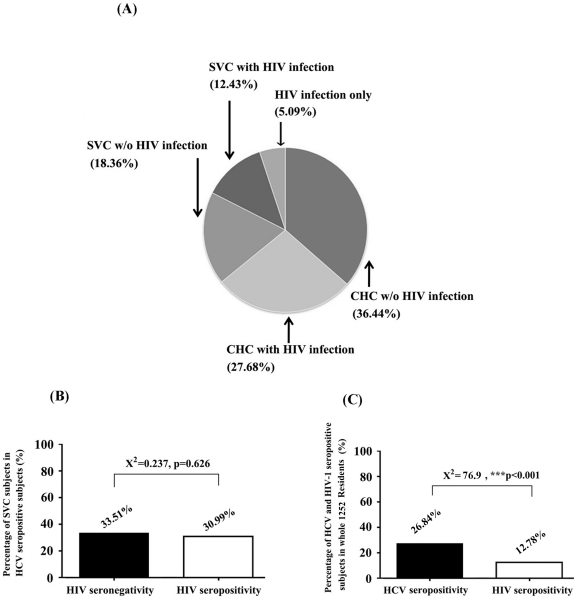
The distributional characteristics of HCV- and/or HIV-infected patients involved in this study. (**A**) 354 HCV seropositive or HIV-1 seropositive participants were divided into 5 different groups (HIV-noninfected CHC, HIV-infected CHC, HIV-noninfected SVC, HIV-infected SVC, HIV infection only) depending on existence of serum HIV-1/HCV antibodies and plasma HCV RNA. The proportions of each group in 354 subjects were indicated. (**B**) Comparison of the percentages of SVC subjects in HCV seropositive subjects between HIV-1 noninfected (*black square*) and HIV-1 infected (*empty square*) patients. (**C**) Percentage of HCV (*black square*) and HIV-1 (*empty square*) seropositive patients in whole 1252 residents. Pearson Chi-Square tests were performed for comparing the percentage between two different groups shown in (**B**) and (**C**). Triple asterisks (***) indicate *P* values below 0.001.

In this study, 354 participants were divided into 5 groups (HIV-noninfected CHC, HIV-infected CHC, HIV-noninfected SVC, HIV-infected SVC, and HIV infection alone) depending on the existence of anti-HCV antibody, anti-HIV antibody, and HCV viral load (Fig. 1A). The clinical data of participants provided by local CDC indicated that more than 90% of the participants infected with HCV and/or HIV were former blood donors (FBDs) between 1990 and 1997, and sex transmission was account for infection of the left individuals. The population of HIV-noninfected CHC individuals, which meant HCV mono-infection as proved by detectable viral load in plasma, accounted for the highest proportion (129/354, 36.44%) among all five groups. More than a quarter of the participants belonged to the population of HIV-infected CHC (98/354, 27.68%), which was a HCV and HIV-1 co-infected group. Both HIV-noninfected SVC (65/354, 18.36%) and HIV-infected SVC (44/354, 12.43%) populations represented HCV seropositive individuals with undetectable HCV viral replication (Fig. 1A), who were considered as having a prior HCV infection and were classified as HCV non-carriers since circulating HCV RNA was maintained at an undetectable level while serum antibody reactions were positive. Based on distributional characteristics of HCV and HIV-1 infection (Fig. 1A), the HCV self-recovery rates in HIV seronegative and HIV seropositive populations, presented as percentage of SVC/HCV seropositivity in Fig. 1B, were calculated. The results indicate that there was no statistical difference (X^2^ = .237, *P* = 0.626) in the percentage of SVC/HCV seropositivity between HIV-1 infected (30.99%) and noninfected (33.51%) populations. The percentage of HCV and HIV seropositivity in all 1252 residents was compared. The data showed that 26.84% of the participants were HCV seropositive, while only 12.78% of the participants were HIV seropositive. There was a significant difference in the seropositive rate between HCV and HIV (X^2^ = 76.9, *P*<0.001, Fig. 1C). The difference between HCV and HIV infection rate may result from a more efficient transmission of HCV than HIV through the blood-borne route as reported previously, or from a higher baseline prevalence of HCV than HIV.

### HIV-infected SVC group had higher levels of serum ALT and AST than HIV-noninfected SVC group while no differences were found between CHC groups with and without HIV infection

**Figure 2:**
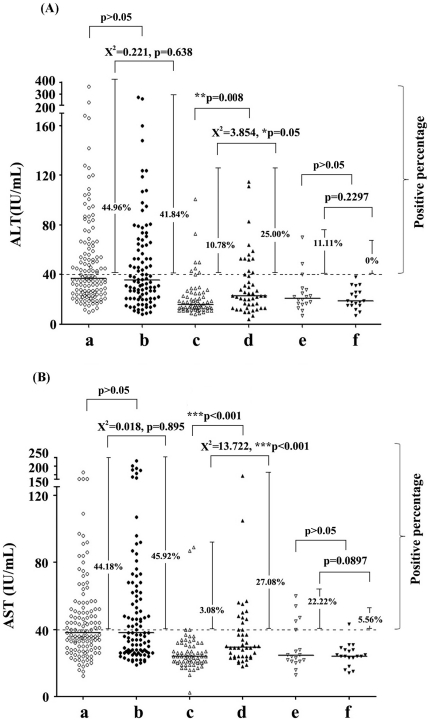
Serum ALT and AST enzymes were comparatively analyzed among five infected groups and healthy controls. (**A**) ALT; (**B**) AST. The percentages of patients with positive ALT or AST values (more than 40 IU/ml) in each group were indicated in the middle beside each group. Median was also shown as a horizontal bar in each group. Group a (○), b(•), c(△), d(▴), e (▽) and f(▾) indicated participants of HIV-noninfected CHC, HIV-infected CHC, HIV-noninfected SVC, HIV-infected SVC, HIV-monoinfected patients and healthy controls respectively. Pearson Chi-Square tests (Yates' correction for continuity was used in certain situations) were performed for comparing the percentages with values above 40 IU/ml and Mann-Whitney U-tests were used for comparing the ALT/AST values between two different groups. Single asterisk (*) indicate P values below 0.05. Double asterisks (**) indicate P values below 0.01 and triple asterisks (***) indicate *P* values below 0.001.

ALT and AST were considered as two critical enzymes for assessing liver function. Normal clinical ranges of serum ALT and AST value were usually designated as no more than 40 IU/ml. We calculated the percentages of subjects with elevated ALT (>40 IU/ml) or AST (>40 IU/ml) values per group. No significant difference of either ALT (X^2^ = 0.221, *P* = 0.638) or AST (X^2^ = 0.018, *P* = 0.895) was found between the HCV mono-infection (HIV-noninfected CHC, 44.96% for ALT and 44.18% for AST) group and the HCV/HIV coinfection (HIV-infected CHC, 41.84% for ALT and 45.92% for AST) group (Fig. 2A and B). Interestingly, there were significant differences of the percentages with values above 40 IU/ml for ALT (X^2^ = 3.854, *P* = 0.05) and AST (X^2^ = 13.722, *P*<0.001) between the HIV-noninfected SVC (10.78% for ALT and 3.08% for AST) and the HIV-infected SVC (25.00% for ALT and 27.08% for AST). The similar significant differences for ALT (*P* = 0.008) and AST (*P*<0.001) absolute values were found between these two subpopulations (Fig. 2A and B). Though percentages of elevated ALT (11.11%) and AST (22.22%) was higher in the HIV mono-infected population than in healthy controls (0% for ALT and 5.56% for AST), there was no statistical difference. This is possibly due to the small population size for HIV mono-infection (n = 18) and healthy controls (n = 19) (Fig. 2A and B). Additionally, no correlation was found between ALT or AST and HCV viral load or between ALT or AST and S/CO ratio of anti-HCV antibody in HIV-noninfected CHC and HIV-infected CHC patients (data not shown).

### S/CO ratios of anti-HCV antibodies were higher in HCV mono-infected than HCV/HIV-1 dual infected individuals

**Figure 3:**
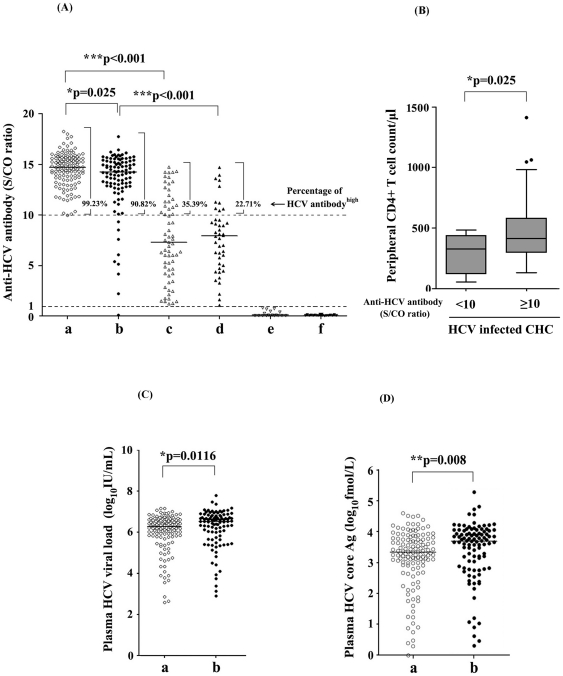
S/CO ratio of anti-HCV antibody, plasma HCV viral load and concentration of plasma HCV core antigen were analyzed among five HCV- and/or HIV-infected groups and healthy controls. (**A**) S/CO ratio of anti-HCV antibody. S/CO ratio was shown as a scatter dot plot graph. Values (S/CO) no less than 10 were considered as HCV antibody^high^ and percentages of patients with HCV antibody^high^ were indicated for each group. (**B**) CD4+ T cells counts in HIV-infected CHC subpopulations based on the level of anti-HCV antibody titer (S/CO). (**C**) Plasma HCV viral load in HIV-1-noninfected CHC and HIV-infected CHC patients. (**D**) Concentration of plasma HCV core antigen in HIV-noninfected CHC and HIV-infected CHC patients. Group a (○), b(•),c(△),d(▴),e (▽) and f(▾) indicated participants of HIV-noninfected CHC, HIV-infected CHC, HIV-noninfected SVC, HIV-infected SVC, HIV-monoinfected patients and healthy controls respectively. Median was shown as a horizontal bar in each group. Mann-Whitney U-tests were performed for statistical comparison between two different groups. Single asterisk (*) indicate *P* values below 0.05. Double asterisks (**) indicate *P* values below 0.01 and triple asterisks (***) indicate *P* values below 0.001.

The S/CO ratios of anti-HCV antibodies measured by the ARCHITECT Anti-HCV System were compared among the five groups mentioned above. Results showed that anti-HCV antibodies levels were much higher in the HCV RNA positive groups than in the HCV RNA negative group, whether with or without HIV-1 co-infection [Fig. 3A, *P*<0.001 for groups (a) HIV-noninfected CHC versus (c) HIV-noninfected SVC; and groups (b) HIV-infected CHC versus (d) HIV-infected SVC]. This result suggests that plasma HCV RNA (positive or undetectable), influenced the level of circulating anti-HCV antibody responses significantly. In addition, there was a significant difference in anti-HCV antibody levels between HCV mono-infection (HIV-noninfected CHC) and HCV/HIV-1 co-infection (HIV-infected CHC) (*P* = 0.025) (Fig. 3A). Anti-HCV Abs S/CO ratios of most of HCV RNA positive hepatitis C patients without HIV-1 infection (99.23%) were more than or equal to 10, while a minority (9 subjects, approximately 10%) of HCV RNA positive HCV/HIV-1 co-infected individuals were tested to carry low S/CO ratios of anti-HCV Abs (less than 10). This result was consistent with the fact that HIV-1 associated immunodeficiency was closely related with an impaired humoral immune response. Also, a significant difference of CD4+T cell counts (*P* = 0.025) was found between subpopulations with anti-HCV antibody titer (S/CO) less than 10 and S/CO no less than 10 (Fig 3B). The CD4+ T cell counts of all nine HIV-infected CHC patients with low anti-HCV antibody titer (S/CO) were less than 500/µl (maximum value of CD4+ T counts was 484/µl in these 9 patients). Interestingly, all these nine patients were positive for both of HCV-RNA and core antigen. This data suggested HIV-associated immunodeficiency had no influence on the sensitivity of HCV core antigen detection.

### Levels of plasma HCV RNA and HCV core antigen were significantly higher in HCV/HIV-1 co-infection than in HCV mono-infection

Plasma HCV viral load and HCV core antigen were measured by Abbott RealTime™ HCV Amplification Kit and Abbott ARCHITECT HCV Ag Assay Kit. Comparisons were only performed for HCV-RNA positive patients (HIV-noninfected CHC and HIV-infected CHC groups) since no detectable HCV viral load was found in SVC and HIV mono-infected patients. It must be pointed out that HCV core antigen was undetectable in all HCV-RNA negative patients while the majority of CHC patients were reactive for HCV core antigen measurement (125/129, 96.90% for HIV-noninfected CHC group and 96/98, 97.96% for HIV-1-infected CHC group). All HCV patients who spontaneously cleared HCV infection were core antigen negative and no patients with positive core antigen while negative HCV-RNA was found in all 354 individuals, which solidly demonstrated the specificity of HCV core antigen test. In addition, four patients who were HCV RNA positive but negative for HCV core antigen testing were found in HIV-noninfected CHC subpopulation. HCV-RNA values of these four patients were 2.6, 2.87, 4.08 and 5.79 log_10_IU/ml respectively. Besides, two patients who were HCV RNA positive but negative for HCV core antigen testing were found in HIV-infected CHC subpopulation and HCV-RNA values of these two patients were 2.90 and 3.13 log_10_IU/ml respectively. This data indicated that patients who were HCV RNA positive but negative for HCV core antigen testing were fewer and usually had very low HCV viral loads. However, there was no evidence supporting that HIV infection may enhance the possibility of this kind of rare event. Statistical analysis showed that both levels of plasma HCV RNA (*P* = 0.0116) (Fig. 3C) and HCV core antigen (*P* = 0.008) (Fig. 3D) were significantly higher in HCV/HIV-1 co-infection (HIV-infected CHC) than in HCV mono-infection (HIV-noninfected CHC). It is conceivable that HCV replicative levels increased faster in HIV-positive subjects than in HCV mono-infected patients [16].

### The concentrations of HCV core antigen in serum were highly correlated with HCV RNA level in HCV mono-infected and HCV/HIV-1 co-infected individuals

**Figure 4:**
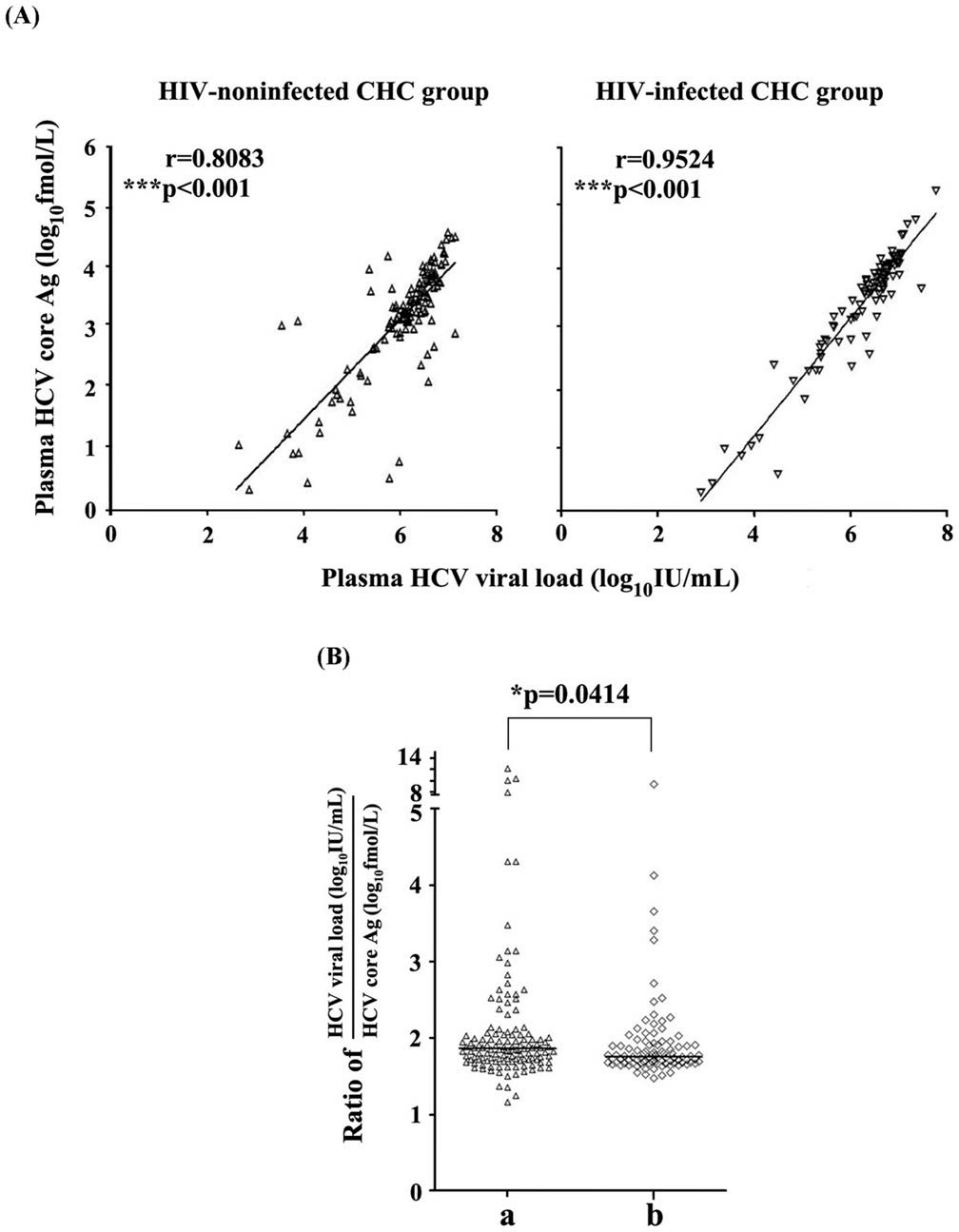
(A) The concentration of HCV core antigen in serum were highly correlated with HCV RNA level in both HIV-noninfected CHC (△)and HIV-infected CHC (▽) patients and (B) ratio of serum HCV viral load to HCV core antigen concentration was significantly higher in HIV-noninfected CHC (a,△) than HIV-infected CHC (b,◊) patients. Spearman's rank-correlation and Mann-Whitney U-tests were performed in (**A**) and (**B**) separately. Single asterisk (*) indicate *P* values below 0.05 and triple asterisks (***) indicate *P* values below 0.001.

In this study, a new commercial chemiluminescent microparticle immunoassay kit for measurement of HCV core antigen was used. HCV core antigen detection has a high sensitivity as mentioned above. We found that 96.90% HIV-noninfected CHC patients and 97.96% HIV-infected CHC patients were reactive to the HCV core antigen measurement. And for the first time, we reported that serum concentrations of HCV core antigen were highly correlated with HCV RNA levels in both HCV mono-infected (r = 0.8083, *P*<0.001) and HCV/HIV-1 co-infected (r = 0.9524, *P*<0.001) patients in this cross-sectional survey (Fig. 4A). Further analysis showed that the correlation was highly similar in both HCV-1b and HCV-2a genotypes CHC patients (data not shown). This suggests that HCV core antigen measurement will be an excellent surrogate for the HCV RNA test to monitor HCV viral replication in clinical application though they focused on completely different targets (protein or nucleic acid) and will provide clinical researchers an alternative method with low cost and easy-manipulation to evaluate the infectious status of HCV infected patients. We further explored whether the ratio of HCV RNA viral load/HCV core antigen concentration correlated with infection status in HCV mono-infection and HCV/HIV-1 co-infection. Interestingly, a significantly higher ratio of HCV RNA viral load/HCV core antigen levels was found in HCV mono-infected patients compared with HCV/HIV-1 co-infected patients (*P* = 0.0414) (Fig. 4B).

### HCV core antigen concentration was negatively correlated with CD4+ T cell counts and S/CO ratio of anti-HCV antibody was positively correlated with CD4+ T cell counts in HIV-infected CHC patients with CD4+ T cell counts less than 1000/µl

**Figure 5:**
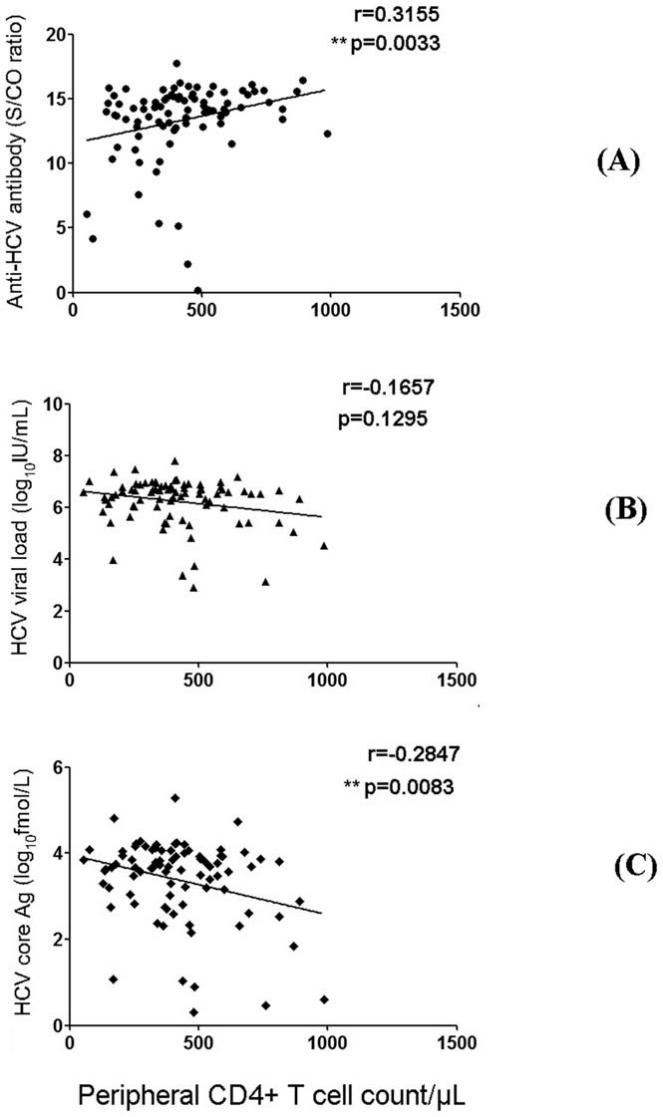
Correlation analysis between anti-HCV Abs response (S/CO ratio)(A), or HCV viral load (B), or HCV core antigen concentration (C) and CD4+ T cell counts displayed in HIV-infected CHC patients with CD4+ T cell counts less than 1000/µl. Spearman's rank-correlation were performed and double asterisks (**) indicated *P* values below 0.01.

We also analyzed the correlation between HCV Abs (S/CO value) and CD4+ T cell counts in patients with CD4 T cell counts less than 1000/µl. Interestingly, a significant positive correlation (r = 0.3155, *P* = 0.0033) was found in HIV-infected CHC patients (Fig. 5A), though no statistical differences were found in the HCV mono-infection (HIV-noninfected CHC) and HIV-noninfected SVC groups (data not shown). As shown in Fig. 5B and C, HCV core antigen concentration was negatively correlate (r = −0.2847, *P* = 0.0083) with CD4+ T cell counts, while no correlation was found between HCV viral load and CD4+ T cell counts in HIV-coinfected CHC patients, though the concentration of serum HCV core antigen was highly correlated with HCV RNA level in both HCV mono-infected and HCV/HIV-1 co-infected individuals as described above. These findings suggested that the fluctuation of HCV core antigen concentration was more sensitive than HCV viral load or HCV viral load was out of synchronization with CD4+ T cell counts in HCV/HIV-1 coinfected individuals. In addition, the effects of HCV RNA or HCV core antigen on peripheral CD4/CD8 T cell counts in HCV mono-infected patients were analyzed and no difference was found (data not shown).

## Discussion

Since the introduction of HAART in 1996, the incidence of most opportunistic illnesses has declined sharply [17], [18], while HCV-related liver disease has become a leading cause of morbidity and mortality in HIV-1-infected individuals. Bica et al. [19] and Monga et al. [20] demonstrated that end-stage liver disease was the leading cause of death in the HIV-1 seropositive population after HAART was introduced. In China, most of HIV-1 infected individuals (including FBDs) have received the first-line HAART regimes consisting of two NRTIs and one NNRTI since 2003. Preliminary data from a survey (still ongoing, unpublished) in our laboratory indicated that HCV infections also accounted for up to 50% of mortality in co-infected FBD individuals.

Several studies [21], [22] have investigated the distribution and correlation of HCV RNA positivity or negativity among HCV-seropositive populations. In our present study, the percentage of HCV RNA negative patients among anti-HCV seropositive patients was a little higher in HIV-1 seronegative (33.51%) than HIV-1 seropositive (30.99%) subpopulations (Fig. 1B) in enrolled participants (more than 90% of subjects having a history of blood donation) while this difference did not reach statistical significance. However, it's hard to draw a conclusion that HCV self-recovery rate has no association with HIV-1 infection since it is difficult to determine if HCV infection occurs before or after HIV-1 infection. If acute HCV infection and self-recovery occur before HIV-1 infection, there should be no association between HCV self-recovery and HIV-1 infection. Since transmission of HCV is more efficient than HIV-1 through unsanitary blood or blood products, it is most possible that HCV self-recovery occurred before HIV-1 infection in the majority of FBDs with HIV-1/HCV coinfection.

A number of cross-sectional studies have investigated the predictors of elevated liver enzymes in HIV-infected patients without HCV infection [23]–[27]. However, the effects of HIV-1 virus *per se* or of HAART on the progression of hepatic damage in HCV infected patients are not well understood. Our results indicated that HIV-associated factors may contribute to the elevated signals (ALT and AST) of liver function since the HIV-infected SVC group showed higher serum ALT (*P* = 0.05) and AST (*P*<0.001) than the HIV-noninfected SVC group (Fig. 2). In our study, 80% (35/44) of HIV-infected SVC patients (including patients with elevated ALT/AST) were taking HAART treatment, regularly or intermittently. HAART regimes consisted of two NRTIs (AZT/ddI or d4T/3TC), and one NNRTI (NVP). Usage of NRTIs might slightly worsen liver function with very low frequency and NVP might be associated with hepatitis on the background of high baseline CD4+ T cell counts [28], [29]. It is understandable that the effect of HIV-associated factors on liver disorders may be mild-to-moderate because no differences were found between CHC groups with and without HIV-1 coinfection. However, it is difficult to definitely conclude which factor played a major role in aggravating liver damage when considering chronic hepato-toxicity related to HAART, incomplete immune recovery, and other possible factors from our current data.

Our data showed that the S/CO ratio of anti-HCV Abs in HCV RNA-positive patients with or without HIV-1 co-infection was significantly higher than that of the patients with HCV RNA seronegativity (Fig. 3A, *P*<0.001), which is consistent with the hypothesis that active HCV intrahepatic replication and stimulation is crucial for the maintenance of higher levels of serum anti-HCV responses. Theoretically, the clearance or extreme decline of HCV intrahepatic replication would result in the gradual decrease of HCV specific antibodies due to the loss of constant stimulation by HCV viral components. Once the virus was cleared away, the titer of serum HCV antibodies would drop gradually and spontaneous seroconversion would finally occur.

Previous studies reported that HCV core Ag quantification by ELISA method such as Lumipulse Ortho HCV Ag (Lumipulse-Ag) (Ortho Clinical Diagnostics) with a detection limit of 50 fmol/l can be used in the various indications of viral load monitoring, including the evaluation of baseline viral load before therapy and the study of early viral kinetics during therapy [30]–[32]. Recently, a highly sensitive assay for HCV core antigen using a fully automated CMIA technique has become commercially available. The reactive cut-off for this assay was set at 3 fmol/l (S/CO = 1.0) for maximum specificity, although the limit of detection has been calculated to be 0.83–1.24 fmol/l [33]. A number of potential clinical uses for this assay have been described, including significantly shorten the diagnostic window period for detection of acute infection, as a tool to monitor the incidence/course of disease in various patient groups such as hemodialysis patients and injection drug users, as a rapid test for confirmation of active infection in anti-HCV positive cases, and as an independent parameter to predict response to treatment [33]. In the present study, both of serum concentration of HCV core antigen and HCV viral load were significantly lower in HCV mono-infection than HCV/HIV-1 co-infection. Of note, quantitation of the CMIA HCV core antigen assay is highly correlated with the corresponding HCV viral load in CHC with or without HIV-1 infection (Fig 4A). Furthermore, HCV core antigen had a detection rate comparable to using HCV RNA testing. In contrast, serum anti-HCV titer in HCV mono-infection was significantly higher than in co-infection. As shown in Fig. 3A, low S/CO ratios (less than 10) of serum anti-HCV antibodies were apparent in a minority of plasma HCV-RNA positive HCV/HIV-1 co-infected patients (approximately 10%). However, this value is notably higher than the percentage with low S/CO ratios (less than 1%) in that of plasma HCV RNA positive HCV mono-infected patients, indicating an increased risk of false negativity of HCV infection only judged by presence of positive anti-HCV antibody in HIV-1 coinfected patients. Fig. 3A indicated that an undetectable anti-HCV antibody (0.09 S/CO) occurred in one HIV-infected CHC patient (HCV viral load: 4.11 log_10_IU/ml; HCV core concentration: 16.03 fmol/l; CD4+ T cells count: 346/µl). As a result, it should be valuable to evaluate the HCV infectious status of an HIV-1 positive population by detecting HCV antigen or RNA. Of note, we demonstrated that a significant positive correlation was found between S/CO ratio of anti-HCV antibodies and peripheral CD4+ T cell counts in HCV/HIV-1 coinfection. Importantly, HCV core antigen concentration was shown to negatively correlate with CD4+ T cell counts (r = −0.2847, *P* = 0.0083), while no correlation was found between HCV viral load and CD4+ T cell counts in HIV-coinfected CHC patients (Fig 5B and C). These findings could be interpreted as a hint that HCV core antigen testing was more sensitive to reflect immune pressure than HCV RNA testing. A possible interpretation was that the half-life of HCV core protein was relatively shorter under the regular human immune condition than under the immunodeficiency condition, whereas the breakdown of serum HCV RNA was less sensitive to immune clearance compared with HCV core protein. This interpretation may be also partially responsible for the higher ratio of HCV RNA to core antigen found in HIV-noninfected CHC patients compared to HIV-infected CHC individuals (Fig 4B).

In this study, we suggested that HCV core antigen could be used as a marker of HCV replication in anti-HCV antibody positive, treatment-naïve population, with or without HIV-1 coinfection. It is well known that the application of HCV core antigen testing on HCV diagnosis has several advantages. First, no sophisticated equipments are needed for HCV core antigen testing and the performance is time-saving in comparison with HCV RNA testing. Second, the expected price of HCV core antigen testing is cheaper than HCV RNA detection. Final, HCV core antigen was shown to be much more stable in serum and plasma than HCV RNA [34], [35]. It was demonstrated that the concentration of HCV core antigen was reproducible and stable even after incubation at room temperature for one week, while the concentration of HCV RNA dropped dramatically after incubation at 25°C for 24 hours [35]. Therefore, HCV core antigen testing may act as an useful alternative marker for quantitative analysis of HCV replication and monitoring anti-HCV therapy.

In total, our findings demonstrated that there were distinctive serological and virological characteristics of serum HCV RNA positive and negative hepatitis C patients with or without HIV-1 coinfection. Importantly, there was an excellent correlation between plasma HCV viral load and the concentration of HCV core protein not only in the HCV mono-infected population but also in the HCV/HIV-1 coinfected population. The HCV antigen assay detected the vast majority of HCV RNA positives with or without HIV-1 infection (97.96% and 96.9%, respectively). Considering HCV, core antigen testing has a comparable sensitivity to HCV RNA qPCR. HCV core antigen concentration, but not HCV RNA level, was negatively correlated with CD4+ T cell counts. Our data suggested that quantitative detection of plasma HCV core antigen may be a novel and alternative indicator of peripheral HCV level than HCV RNA level when evaluating the association between HCV replication and host immune status in HCV/HIV-1 coinfected patients.

## Supporting Information

Table S1 **Characteristics of 354 patients with HCV and/or HIV-1 seropositivity enrolled in the study.**
(DOC)
