# Supplementary material for: A Higher Correlation of HCV Core Antigen with CD4+ T Cell Counts Compared with HCV RNA in HCV/HIV-1 Coinfected Patients
Source: PLoS One. 2011 Aug 12;6(8):e23550. doi: 10.1371/journal.pone.0023550 (PMC3155566; doi:10.1371/journal.pone.0023550)
Supplement: Table S1 — Characteristics of 354 patients with HCV and/or HIV-1 seropositivity enrolled in the study. (DOC) [file pone.0023550.s001.doc]

Table S1. Characteristics of 354 patients with HCV and/or HIV-1 seropositivity enrolled in the study

| **Variable** | **HIV-noninfected CHC (n=129)** | **HIV-infected CHC (n=98)** | **HIV-noninfected SVC (n=65)** | **HIV-infected SVC (n=44)** | **HIV infected only (n=18)** |
| --- | --- | --- | --- | --- | --- |
| **Sex** |  |  |  |  |  |
| **Female** | 61(47) | 53(54) | 51(78) | 24(55) | 10(56) |
| **Male** | 68(53) | 45(46) | 14(22) | 20(45) | 8(44) |
| **Age,median years(IQR^a^)** | 51(42-59) | 43(39-52) | 46(39-55) | 44(41-52) | 41(34-47) |
| **CD4+ cell count,median cells/ul(IQR)** | 812(589-1045) | 409(288-550) | 875(684-957) | 385(251-566) | 404(264-678) |
| **CD8+ cell count,median cells/ul(IQR)** | 541(444-859) | 931(667-1187) | 623(412-894) | 972(706-1238) | 842(711-1011) |
| **Anti-HCV,median S/CO (IQR)** | 14.70(13.69-15.59) | 14.18(12.83-15.23) | 7.29(4.58-10.98) | 7.49(4.45-9.30) | 0.09(0.07-0.39) |
| **HCV RNA,median logIU/ml(IOR)** | 6.27(5.83-6.60) | 6.53(5.87-6.79) | 0 | 0 | 0 |
| **HCV Ag,median logfmol/L(IQR)** | 3.34(2.92-3.77) | 3.69(2.93-4.02) | 0.14(0-0.42) | 0.28(0-0.59) | 0 |
| **HCV genotype (2a/1b)** | 45/84 | 56/42 | NA^h^ | NA | NA |
| **Biochemistrial analysis** |  |  |  |  |  |
| **ALT^b^,median IU/ml(IQR)** | 37(25-61) | 36(21-65) | 16(13-25) | 20(15-41) | 21(17-27) |
| **AST^c^,median IU/ml(IQR)** | 38(30-52) | 38(27-62) | 24(21-30) | 30(24-45) | 25(21-37) |
| **γ-GT^d^,median IU/ml(IQR)** | 19(16-28) | 35(20-73) | 16(13-21) | 29(16-57) | 25(19-48) |
| **ALP^e^,median IU/ml(IQR)l** | 83(66-114) | 112(87.25-152.5) | 87(61-113) | 104.5(91.75-115.75) | 87.5(66.5-115) |
| **Albumin,median g/L(IQR)** | 44.1(40.3-47.2) | 44.85(41.9-48.9) | 43.4(40.1-47.6) | 44.8(41.93-48.55) | 44.45(39.85-48.58) |
| **Total protein,median g/L(IQR)** | 76.8(73.9-79.6) | 77.4(74.4-82.58) | 75.1(72-78.2) | 76.4(73.98-79.6) | 75.85(70.73-80.55) |
| **Total bilirubin,median μmol/L(IQR)** | 13.84(11.11-16.3) | 12.76(10.75-16.48) | 13.13(10.45-15.65) | 14.8(11.45-16.59) | 13.82(10.63-15.63) |
| **Direct bilirubin,median μmol/L(IQR)** | 4.24(3.35-5.16) | 4.43(3.36-5.48) | 4.62(3.34-5.22) | 4.12(3.20-5.20) | 5.74(4.32-5.97) |
| **Hemoglobin,median g/L(IQR)** | 143(128-154) | 126.5(112.75-146) | 131(114-144) | 121.5(111-142) | 117.5(19.5-124.5) |
| **BMI^f^,median value(IQR)** | 23.28(21.20-25.53) | 22.01(20.71-24.32) | 23.31(21.85-26.43) | 23.12(20.71-23.12) | 23.10(21.72-24.50) |
| **Hepatitis** | 18(14) | 10(10) | 4(6) | 8(18) | 1(6) |
| **Fatty liver** |  |  |  |  |  |
| **Low-grade** | 8(6) | 18(18) | 2(3) | 7(16) | 2(11) |
| **Medium-grade** | 0 | 1(1) | 2(3) | 7(16) | 0 |
| **Cirrhosis** | 2(2) | 0 | 0 | 0 | 0 |
| **Liver cancer** | 0 | 0 | 0 | 0 | 0 |
| **Hypertension** | 16(12) | 15(15) | 14(22) | 8(18) | 1(6) |
| **Diabetes** | 3(2) | 1(1) | 1(2) | 2(4) | 0 |
| **Hyperlipidemia** | 16(12) | 6(6) | 7(11) | 5(11) | 2(11) |
| **Hepatic nephropathy** | 2(2) | 8(8) | 1(2) | 3(7) | 0 |
| **Smoking status** |  |  |  |  |  |
| **Never** | 71(55) | 55(56) | 54(83) | 27(61) | 12(67) |
| **Former** | 5(4) | 4(4) | 0 | 2(4) | 0 |
| **Current** | 53(41) | 39(40) | 11(17) | 15(34) | 6(33) |
| **Alcohol consumption** |  |  |  |  |  |
| **None** | 99(77) | 72(73) | 57(88) | 35(80) | 15(83) |
| **Mean g/day(IQR)** | 97.31(43.18-349.86) | 267.95(52.71-267.95) | 66.08(16.38-255.62) | 63.12(12.33-475.62) | 299.18(193.42-596.71) |
| **HARRT^g^** |  |  |  |  |  |
| **Occasional** | NA | 8(8) | NA | 3(7) | 2(11) |
| **Intermittent** | NA | 12(12) | NA | 3(7) | 2(11) |
| **Regular** | NA | 73(74) | NA | 36(82) | 14(78) |
| **Unclear (Percentage)** | NA | 5(6) | NA | 2(4) | 0(0) |
| **Duration,median years(IQR)** | NA | 6.5(4.5-7.4) | NA | 6.5(5.5-7.0) | 6.5(5.8-6.9) |

**NOTE.** Data are no.(%) of patients, unless otherwise indicated. a: IQR, interquartile range; b: ALT, alanine aminotransferase; c: AST, aspartate aminotransfearse; d: γ－GT, γ－glutamyltransferase; e: ALP, alkaline phosphatase; f: BMI, body mass index, calculated as the weight in kilograms divided by the square of height in meters; g: HAART, highly active antiretroviral therapy; h: NA, non available. The detection limit of the HCV antigen assay is 3 fmol/L (log 3 = 0.477).
